# Selective deletion of the soluble Colony-Stimulating Factor 1 isoform *in vivo* prevents estrogen-deficiency bone loss in mice

**DOI:** 10.1038/boneres.2017.22

**Published:** 2017-11-14

**Authors:** Gang-Qing Yao, Nancy Troiano, Christine A Simpson, Karl L Insogna

**Affiliations:** 1 Department of Internal Medicine, Yale University School of Medicine, New Haven, USA; 2 Department of Orthopaedics, Yale University School of Medicine, New Haven, USA

## Abstract

Neutralizing CSF1 *in vivo* completely prevents ovariectomy (OVX)-induced bone loss in mice. There are two isoforms of CSF1, soluble (sCSF1), and membrane-bound (mCSF1), but their individual biological functions are unclear. It had been previously reported that mCSF1 knockout (K/O) and wild type (Wt) female mice experience the same degree of bone loss following OVX. In Wt mice the expression of sCSF1 was elevated fourfold in skeletal tissue following OVX while expression of mCSF1 was unchanged. To examine the role of sCSF1 in OVX-induced bone loss, mice were engineered in which sCSF1 was not expressed but expression of mCSF1 was unaffected (sCSF1 K/O). Isoform-specific reverse transcription PCR confirmed the absence of transcripts for sCSF1 in bone tissue isolated from these animals and no circulating CSF1 was detected by ELISA. Surprisingly, there were no significant differences in bone mineral density (BMD) between sCSF1 K/O mice and Wt controls as assessed by dual-energy X-ray absorptiometry and micro-CT. However, one month after OVX, femoral, spinal and total BMD had declined by 11.2%, 8.9%, and 8.7% respectively in OVX-Wt animals as compared to Sham-OVX. In contrast OVX sCSF1 K/O mice showed changes of +0.1%, −2.4%, and +2.3% at the same 3 sites compared to Sham-OVX sCSF1 K/O mice. These data indicate important non-redundant functions for the two isoforms of CSF1 and suggest that sCSF1, but not mCSF1, plays a key role in estrogen-deficiency bone loss.

## Introduction

Alternative splicing is the major source of proteome diversity.^1,2^ Mutations in splicing sites and changes in the relative expression levels of isoforms have been implicated in the pathogenesis of human diseases.^3,4^

Colony-stimulating factor 1 (CSF1) is one of two cytokines absolutely required for osteoclastogenesis. Multiple human CSF1 mRNA species (4.0, 3.0, 2.3, 1.9, and 1.6 kb) are transcribed from the *CSF1* gene,^5–9^ and molecular cloning of cDNAs derived from these transcripts has demonstrated that the size differences are due to alternative splicing in exon 6 and the alternative use of the 3′-end of exons 9 and 10.^6–8^ The 1.6 and 3.0 kb CSF1 cDNAs give rise, by alternative splicing, to a short exon 6 in which the site for proteolytic cleavage of the CSF1 precursor has been spliced out. Thus, the products of the 1.6- and 3.0 kb cDNAs are cell-surface (membrane-bound) glycoproteins, which are slowly and inefficiently released from the cell surface by extracellular proteolysis.^9,10^ By contrast, the products of the 1.9, 2.3, and 4.0 kb cDNAs have intermediate or long versions of exon 6 in which the proteolytic cleavage site is intact, giving rise to a soluble, rapidly secreted growth factor.^11,12^ Similar findings have been reported for the murine *CSF1* gene.^13,14^

The role of these two isoforms in pathologic states of bone remodeling has not been extensively studied. Friel *et al* reported that mCSF1 supported long-term proliferation of hematopoietic precursor cells, as well as self-renewal of this population.^15^ In contrast, sCSF1 did not support a self-renewing population of progenitors but rather drove cells to differentiate into monocytes and macrophages. Douglass *et al*.^16^ reported that mCSF1 promotes prolonged signal transduction in bone-marrow-derived macrophages. Perhaps related to this latter observation, macrophages can kill mCSF1-positive tumor cells^17,18^ but not sCSF1-positive cells.^19^ We have shown that human and mouse osteoblasts also express mCSF1, that PTH and TNF increase expression of the mRNA and protein product, and, most importantly from a physiologic standpoint, that mCSF1 supports the formation of osteoclast-like cells in a dose-dependent manner *in vitro* and at physiologic concentrations acts synergistically with sCSF1 to induce osteoclast formation.^20,21^ Further, we have found that selective expression of mCSF1 alone in osteoblasts rescues the osteopetrotic phenotype of *op/op* mice.^22^ Since osteoblast-derived sCSF1 does not induce osteoclast-like cells (OCLs) formation in the absence of cell-cell contact, even if RANKL is added,^23^ it appears that mCSF1 may be required for normal osteoclastogenesis.

Kimble *et al*.^24^ reported that estrogen-withdrawal was associated with increased levels of IL-1 and TNF in bone marrow, which induced the formation of a stromal cell population producing high levels of sCSF1. The emergence of this population of stromal cells correlated with increased osteoclastogenesis. The same group subsequently reported that stromal cells from estrogen-deficient animals demonstrated enhanced phosphorylation of the nuclear transcription factor Egr-1, which causes reduced binding of Egr-1 to the transcriptional activator Sp-1 and higher levels of free Sp-1.^25^ Increased levels of free Sp-1 induce enhanced transactivation of the *CSF1* gene, leading to increased CSF1 production in the estrogen-deficient state.^25^ The relevance of this molecular pathway for estrogen-deficiency bone loss is supported by the observation that Egr-1 knockout animals demonstrate increased basal rates of bone resorption that do not increase further following ovariectomy (OVX).^26^ A neutralizing antibody to CSF1 restored rates of bone resorption to normal in these animals and also completely prevented ovariectomy-induced bone loss in Wt animals.^26^ In the aggregate, these data strongly suggest an important role for sCSF1 in estrogen-mediated bone loss. In contrast to these findings, using semi-quantitative reverse transcription PCR, Flanagan and co-workers have reported that estrogen-withdrawal selectively upregulates expression of the cell-surface CSF1 isoform in rat bone marrow cultures.^27,28^

We have selectively deleted mCSF1 in mice and reported that, 1 month after surgery, 5-month-old mCSF1 K/O and Wt female mice experienced the same degree of bone loss following OVX. OVX induced a significant fourfold increase in the expression of the sCSF1 in the bones of Wt mice while expression of mCSF1 was unchanged.^29^ These findings indicate that mCSF1 does not appear to be required for estrogen-deficiency bone loss, but sCSF1 could play a key role in this pathologic process. To explore the role of sCSF1 in estrogen-deficiency bone loss, we have generated isoform-selective sCSF1 K/O mice and report our findings in this model.

## Materials and methods

### Generation of sCSF1 K/O mice

**Figure 1:**
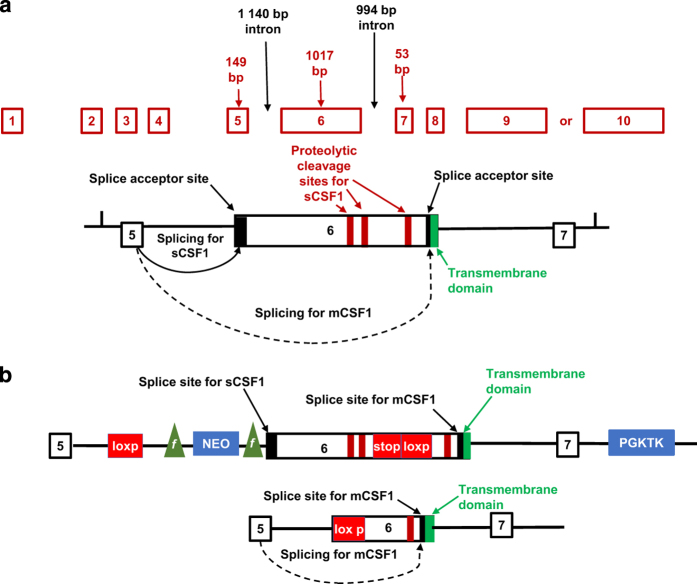
Strategy for generating sCSF1 K/O mice. (**a**) Splicing pattern for the *CSF1* gene. (**b**) The targeting construct for deletion of sCSF1 introduces a stop codon followed by a lox-p site into exon 6 at a position 3′ to the splicing site for sCSF1. The stop codon is designed to include all three reading frames. The Neo cassette with flanking frt sites (lox-p-frt-Neo-frt) is introduced into intron 5. Excision of the Neo cassette with flp recombinase leads to a CSF1 allele that contains two lox-p sites, one in intron 5, and one which is downstream of the splice site for sCSF1. In animals bearing two of these floxed alleles (that is, before cre-mediated recombination) both isoforms of CSF1 will be generated. Splicing to the sCSF1 splice site will lead to generation of an mRNA that includes the sequences for the proteolyitc cleavage sites for sCSF1, followed by a stop codon. The protein product of this mRNA will yield normal mature sCSF1 since it contains the appropriate signals for proteolytic cleavage with the surrounding sequences. Recombination with cre leads to selective deletion of the splice acceptor site for the sCSF1 isoform. This leaves the splice acceptor site for mCSF1 intact. Thus, a mature functional mCSF1 will be generated, but no sCSF1.

The normal splicing patterns of the CSF1 gene that result in expression of both the sCF1 and mCSF1 isoforms are shown in Figure 1a. The targeting construct for deletion of sCSF1 (Figure 1b) introduced a stop codon followed by a lox-p site into exon 6 at a position 3′ to the splice site and proteolytic cleavage sites for sCSF1. The stop codon was designed to include all three reading frames. A Neo cassette with flanking frt sites (lox-p-frt-Neo-frt) was introduced in intron 5.

The linearized targeting vector was electroporated into W9.5 ES cells by the Animal Genomics Services at Yale. Drug-resistant clones were screened by PCR using the primer sets (Forward: 5′-GAAGCCCCTCAGTCTTTCCT-3′ and Reverse: 5′- GCCTGAAGAACGAGATCAGC-3′) and confirmed by Southern analysis. One correctly targeted ES clone was selected for blastocyst injection. The resulting chimeras were mated with Flp-e deleter females to generate sCSF1^flox^^/^^flox^ mice. In animals bearing two of these floxed alleles both isoforms of CSF1 are generated. Splicing to the sCSF1 splice site will lead to generation of an mRNA that includes the sequences for the proteolytic cleavage sites for sCSF1 followed by a stop codon. The protein product of this mRNA will yield normal mature sCSF1 since it contains the appropriate signals for proteolytic cleavage as well as the surrounding sequences.

sCSF1^flox^^/^^flox^ mice were then bred to transgenic animals expressing cre under the control of the human beta actin gene promoter to generate sCSF1^flox^^/^^+^ Cre^+/−^ mice.

Breeding sCSF1^flox/+^ Cre^+/^ mice with sCSF1^flox/flox^ animals generated mice either expressing cre recombinase, ie sCSF1 K/O mice, or mice not expressing cre, which served as Wt controls. The Yale Animal Care and Use Committee approved the use of animals in this study.

### Measurement of sCSF1, mCSF1 transcripts and protein

PCR: mCSF1 and sCSF1 were detected by reverse transcription PCR. The forward primer for both mCSF1 and sCSF1 is: 5′-ACCAACTGGGACGATATGGAGAAGA-3′; and the reverse primers respectively are for mCSF1: 5′-GCTTGAGGGCAAGAGAAGTACC-3′; and for sCSF1: 5′-ATCCTTTCTATACTGGCAG-3′.

ELISA: mCSF1 expression on murine osteoblast plasma membranes from sCSF1 K/O and Wt mice was quantified using a CSF1 ELISA kit from R&D Systems (Minneapolis, MN, USA) as described previously.^22^ Primary murine osteoblasts were prepared from calvariae of 4 to 7-day-old mice by serial collagenase-dispase digestion.^22^ Cells were grown in Î±-minimum essential medium, supplemented with 10% FBS, penicillin, streptomycin, l-glutamine, and 20 mmol·L^−1^ HEPES. Plasma membranes were extracted as described previously.^21^ Briefly, primary cultured mouse osteoblasts were washed with cold PBS and incubated with lysis buffer (10 mmol·L^−1^ Tris-HCl, pH 7.4, 10 mmol·L^−1^ NaCl, 1.5 mmol·L^−1^ MgCl_2_ 1 mmol·L^−1^ dithiothreitol, 0.5 mmol·L^−1^ phenylmethylsulfonyl fluoride, 0.5 μg·mL^−1^ pepstatin A, and 0.5 μg·mL^−1^ leupeptin) and allowed to swell for 15 min on ice. The cells were disrupted using a ground glass homogenizer in an ice bath, and the homogenate was centrifuged at 1 000 *g* for 10 min to remove nuclear and cellular debris. The supernatant was overlaid on a 35% sucrose solution and centrifuged for 60 min at 20 000 *g*. The plasma membranes, which were found in a single band at the interface of the supernatant and sucrose, were collected and centrifuged for 60 min. at 100 000 *g*. The pellets were resuspended and stored at −70 °C until assayed. The concentration of plasma membrane protein was determined using a commercially available kit and the manufacturer’s recommended protocol (Bio-Rad, Hercules, CA, USA).

To measure circulating levels of CSF1, blood was collected by cardiac puncture from K/O mice and Wt littermates, allowed to clot, spun and sera isolated. Circulating levels of CSF1 in these sera were measured using the same murine CSF1 ELISA kit described above.

### BMD measurements

*In vivo* bone mineral density (BMD) measurements were performed by dual-energy X-ray absorptiometry (DXA) using a PIXImus densitometer (Lunar Corporation, Madison, WI, USA). Anesthetized mice (ketamine, 30 mg·kg^−1^ body-Wt and xylazine, 3 mg·kg^−1^ body-Wt given intraperitoneally) were placed in the prone position and scans performed with a 1.270-mm-diameter collimator, 0.762-mm line spacing, 0.380-mm point resolution and an acquisition time of 5 min. The spine window is a rectangle spanning a length of the spine from T1 to the beginning of the sacrum. The femur window encompasses the entire right femur of each mouse. The coefficient of variation for total-body BMD is ~1.5%.

#### Micro-CT

Femurs were stripped of soft tissue and stored in 70% EtOH at 4 °C. Specimens were analyzed in 70% EtOH by cone beam microfocus X-ray computed tomography using a Scanco μCT-35 instrument (Scanco, Brutissellen, Switzerland). Images were acquired at 55 kVp, with an integration time of 500 ms and an isometric voxel size of 6 mm. Segmentation of bone from marrow and soft tissue was performed in conjunction with a constrained Gaussian filter (support=1; 3×3×3 voxel window; *σ*=0.8) to reduce noise, applying density thresholds of 250 and 420 for the trabecular and cortical compartments of the femur, respectively. Volumetric regions for trabecular analysis were selected within the endosteal borders of the distal femoral metaphysis to include the secondary spongiosa located 1 mm from the growth plate and extending 1 mm proximally. Cortical morphometry was quantified and averaged volumetrically through 233 serial cross-sections (1.4 mm) centered on the diaphyseal midpoint between proximal and distal growth plates.

### Bone histomorphometry

Histomorphometry was performed as previously reported.^30–32^ At the time of sacrifice, the tibiae were removed, stripped of soft tissue, and fixed in 70% ethanol. Tibiae were then dehydrated through graded ethanol, cleared in toluene, infiltrated with increasing concentrations of methylmethacrylate, and embedded in methylmethacrylate according to previously described methods.^30,31^ Analyses were performed on 5 μmol·L^−1^ thick sections stained with toluidine blue, pH 3.7 using a Nikon microscope interfaced with the Osteomeasure system software and hardware (Osteometrics, Atlanta, GA, USA). Measurements were obtained in an area of cancellous bone that measured ~2.5 mm^2^, containing only secondary spongiosa, and located 0.5–2.5 mm distal to the epiphyseal growth cartilage. Longitudinal sections (5 μm thick) taken in the frontal plane through the cancellous bone of the proximal tibia were prepared with a Leica RM2165 microtome, mounted on chrom-alum-coated glass slides, and stained with toluidine blue, pH 3.7. All indices were defined according to the American Society of Bone and Mineral Research histomorphometry nomenclature.^33^

### Ovariectomy

Ovariectomy (OVX) was accomplished through a paralumbar incision in anesthetized animals as previously reported.^34^ Each ovarian bursa opposite each ovarian hilum was incised, the ovarian hilum exposed and clamped, and the ovary was removed. Sham ovariectomy animals underwent anesthesia and the paralumbar incision without removal of the ovaries. Four weeks after ovariectomy, BMD was determined by DXA.

### Statistical analyses

Two-way analysis of variance with, when appropriate, *post hoc* testing was used to analyze the DXA data. Non-parametric Mann–Whitney test was used to analyze the micro-CT data. Graph Pad Prism v7.0 (La Jolla, CA, USA) was used for these analyses.

## Results

### Confirmation of isoform-selective deletion of sCSF1

**Figure 2:**
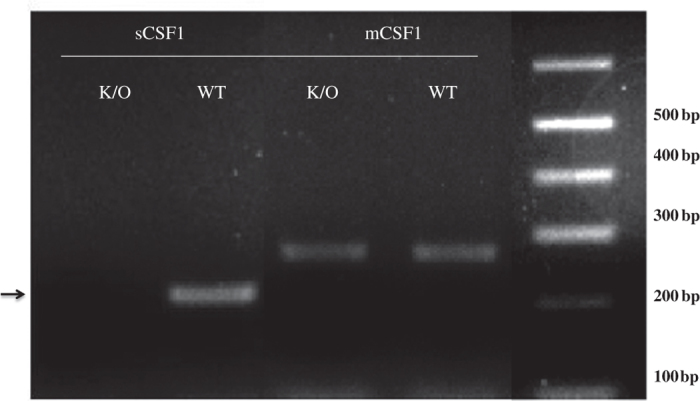
Determination of sCSF1 RNA expression levels in sCSF1 K/O mice. Isoform-specific RT-PCR of bone RNA demonstrates the absence of sCSF1 transcripts in sCSF1 K/O mice. Arrow indicates the expected amplicon, which is present in Wt but not in K/O animals. K/O, knockout; RT-PCR, reverse transcription PCR.

To determine whether our molecular strategy resulted in isoform-selective deletion of sCSF1, RNA was isolated from tibiae of eight-week-old Wt and sCSF1 K/O mice. Isoform-specific quantitative reverse transcription PCR confirmed the absence of sCSF1 transcripts in sCSF1 K/O mice. However, both Wt and sCSF1 K/O mice expressed the transcript for mCSF1 (Figure 2). Circulating levels of CSF1 from 20 Wt and 20 sCSF1 K/O animals were determined. The mCSF1 concentrations in isolated osteoblast membranes prepared from five Wt and five sCSF1 K/O mice were also examined and no significant difference was observed between Wt and sCSF1 K/O mice (*P*>0.05). The results are summarized in Table 1.

### sCSF1 K/O mice have normal BMD

**Figure 3:**
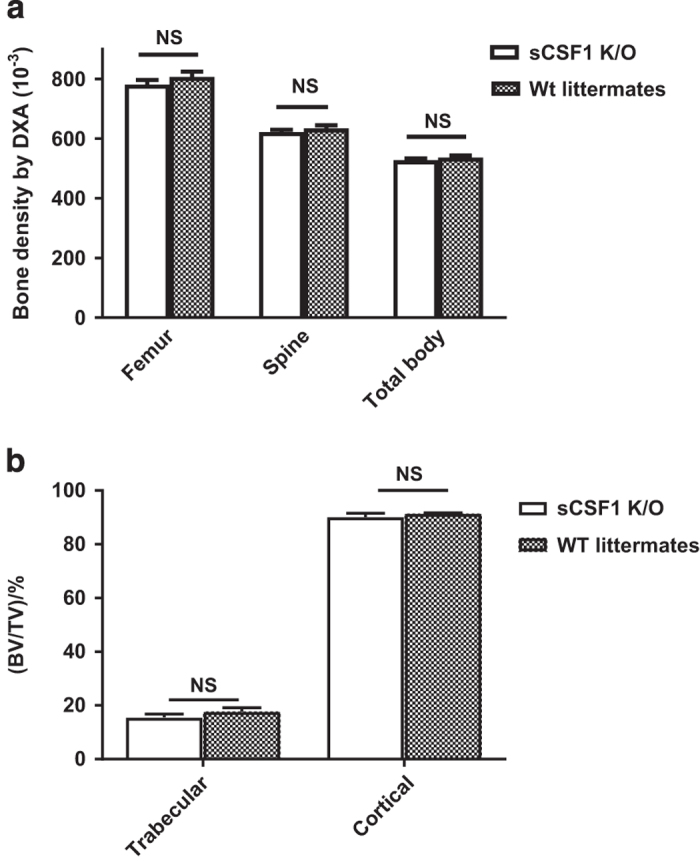
sCSF1 K/O mice have normal bone density. (**a**) Femur, spine, and total-body BMD determined by DXA in 37 Wt (17 male and 20 female) and 48 K/O (19 male and 29 female) mice. (**b**) Femoral trabecular and cortical bone mass assessed by micro-CT in sCSF1 K/O animals (10 male and 10 female animals of each genotype). Results are expressed as M±s.e.m. and analyzed by two-way ANOVA for the data in **a** and by Mann–Whitney for the data in **b**. ANOVA, analysis of variance; BMD, bone mineral density; DXA, dual-energy X-ray absorptiometry; K/O, knockout; M, mean; NS, not significant; Wt, wild type.

Femur, spine, and total-body BMD determined by DXA are shown in Figure 3a: Femur: Wt (0.080 6±0.001 8) vs K/O (0.078 1±0.001 7) g·cm^−2^; Spine: Wt (0.063 4±0.001 1) vs K/O (0.062 2±0.000 8) g·cm^−2^; Total body: Wt (0.052 7±0.000 7) vs K/O (0.052 7±0.000 7) g·cm^−2^. There were no significant differences in bone density at any site between sCSF1 K/O mice and controls when analyzed by two-way analysis of variance. When analyzed by micro-CT there were again no significant differences noted between sCSF1 K/O and control mice: Data are shown in Figure 3b: Trabecular BV/TV: Wt (17.8±0.1)% vs K/O (15.6±0.01)%; Cortical BV/TV: Wt (91.5±0.02)% vs K/O (90.2±0.1)%.

### The genetic absence of sCSF1 attenuates estrogen-deficiency bone loss

**Figure 4:**
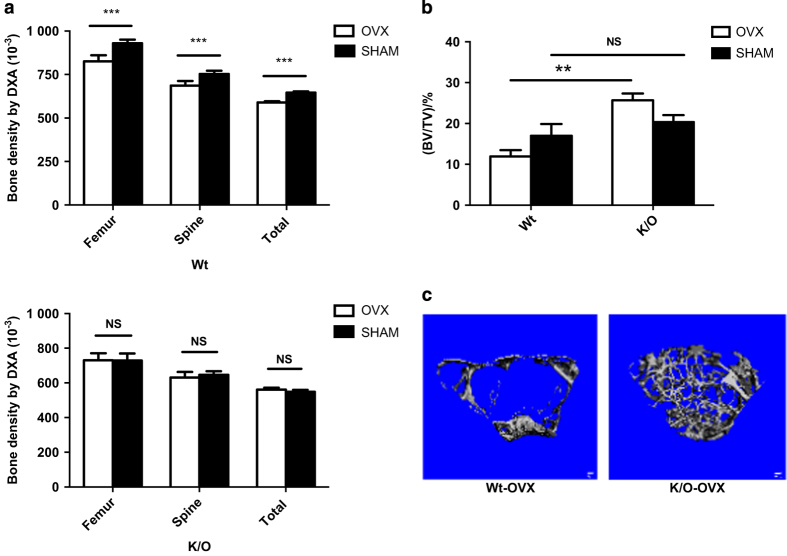
sCSF1 K/O mice are protected against estrogen-deficiency bone loss. (**a**) Mean BMD values determined by DXA in Wt mice (upper panel) and K/O mice (bottom panel) following sham-OVX or OVX. (**b**) Femoral trabecular BV/TV assessed by micro-CT. (**c**) 3D reconstructions of micro-CT images of diaphyseal bone in Wt-OVX and K/O-OVX. Note the greater amount of trabecular bone in the K/O-OVX animals. Results are expressed as M±s.e.m. and analyzed by two-way ANOVA for the data in **a** and by Mann–Whitney for the data in **b**. ANOVA, analysis of variance; BMD, bone mineral density; K/O, knockout; M, mean; NS, not significant; OVX, ovariectomy, ***P<0.000 1, **P=0.02.

To examine the impact of the genetic absence of sCSF1 on estrogen-deficiency bone loss, 19–22-week-old sCSF1 K/O and control female mice underwent OVX or sham-OVX. The results from two independent experiments are summarized in Figure 4 representing data from a total of 69 mice. Mean BMD values in Wt mice were: femur: OVX (0.082 6±0.003 4) vs sham-OVX (0.092 9±0.002 0) g·cm^−2^; spine: OVX (0.068 6±0.002 6) vs sham-OVX (0.075 3±0.001 8)g·cm^−2^; total body: OVX (0.059 0±0.000 6) vs sham-OVX (0.065 0±0.000 6)g·cm^−2^. In K/O mice mean BMD were: femur: OVX (0.073 0±0.004 1) vs sham-OVX (0.073 0±0.004 0) g·cm^−2^; spine: OVX (0.063 1±0.003 3) vs sham-OVX (0.064 7±0.002 1) g·cm^−2^; total body: OVX (0.056 2±0.001 2) vs sham-OVX (0.054 9±0.001 2) g·cm^−2^. Spinal bone density declined by 8.9% in control animals, while it only declined by 2.3% in the sCSF1 K/O mice. At the femur, BMD declined by 11.2% in controls but there was little change in BMD at this skeletal site in the sCSF1 K/O mice. Total BMD fell by 8.7% in the control animals, but increased by 2.3% in the K/O animals (Figure 4a). The differences in the degree of bone loss at the spine, femur, and total-body BMD were statistically significant based on genotype by two-way analysis of variance. The results of micro-CT analyses of femoral BMD were consistent with the above DXA findings. In particular, trabecular BV/TV in the OVX-Wt mice declined by 29% but increased 25% in OVX-K/O mice and the difference in mean BMD after OVX was statistically significantly different based on genotype (Figure 4b and c).

### OVX does not increase osteoclast number in sCSF1 K/O mice

To explore the cellar basis for the differential response to OVX in Wt and sCSF1 K/O mice, histomorphometric analyses were performed on femoral trabecular bone in both groups of animals 1 month after OVX (Table 2). The numbers of osteoblasts, whether expressed referent to bone surface, osteoid surface, total area or bone perimeter were similar in sCSF1 K/O and control mice. In contrast, OVX-K/O mice had significantly lower numbers of osteoclasts compared to the number of osteoclasts in OVX-Wt mice whether referent to bone surface, total area or bone perimeter.

## Discussion

Although, CSF1 is required for osteoclastogenesis, the relative importance of the two CSF1 isoforms in mediating osteoclastogenesis, as well as their individual roles in skeletal homeostasis, remain incompletely understood.

As noted in the Introduction, selective deletion of mCSF1 in mice resulted in an increase in bone mass but did not affect the degree of bone loss following ovariectomy. In contrast to those studies, the current work demonstrates that sCSF1—at least in mice—is dispensable for maintaining normal skeletal mass and normal rates of bone turnover. Thus, there was no difference in bone mass in the sCSF1 K/O mice as compared to littermate controls, whether assessed by DXA or by micro-CT. The data are surprising in so far as sCSF1 is generally considered the major and most important form of CSF1, since it is the principal contributor to the circulating form of the molecule. However, CSF1 can be made by a wide variety of cells, so ascribing unique importance of the sCSF1 isoform to bone, based on its circulating concentrations, may be an over-simplification.

While our findings suggest that sCSF1 is not required for normal skeletal acquisition, this isoform may play a role in inflammatory arthritides. Dalbeth *et al*.^35^ have reported that serum levels of CSF1 are markedly higher in patients with psoriatic arthritis than in patients with psoriasis who do not have joint inflammation. CSF1 and its receptor c-fms are more elevated in in the synovium and synovial fluid of patients with rheumatoid arthritis, compared to those with osteoarthritis.^36,37^ An anti-CSF1 antibody, an antibody to c-fms, and the c-fms inhibitors Ki20227 and GW2580, have all been shown to significantly reduce the severity of joint disease in a murine model of inflammatory arthritis.^36–40^

In earlier work, we reported the results of selectively overexpressing either sCSF1, mCSF1 or both isoforms in osteoblasts *in vivo* using transgenic technology. We reported that targeted overexpression of human sCSF1, human mCSF1 or both (s/mCSF1) in osteoblasts *in vivo* results in significant osteopenia in all three genotypes.^41^ When analyzed by sex, sCSF1 transgenic and m/sCSF1 double-transgenic female animals, but not mCSF1 transgenic female mice, were found to have lower bone mass than their male littermates.^41^ By breeding CSF1 isoform-selective transgenic mice to an *op/op* background, mice were generated in which a single CSF1 isoform was the only source of the cytokine (sCSF1*op/op* and mSCF1*op/op*). Interestingly, when compared to sham-OVX mice of the same genotype, OVX in sCSF1*op/op* mice led to a greater loss of spinal BMD (22.1%) than was seen in either mCSF1*op/op* mice (12.9%) or in Wt animals. We also demonstrated that there is a selective increase in the expression of sCSF1, but not mCSF1, in Wt osteoblasts cultured in estrogen-deficient media. This is consistent with earlier work by Kimball *et al.*, described in the Introduction, indicating that expression of sCSF1 was increased in stromal cells isolated from OVX mice.

This, then leads to the central hypothesis tested in this study, which is that the selective deletion of sCSF1 should attenuate estrogen-deficiency bone loss. Indeed, that is precisely what was observed. Although bone loss was not completely abrogated in the spine it was markedly attenuated at that site, and there was essentially no bone loss in the femur or total body. Thus, the spinal bone density declined by 8.9% in the control animals as compared to 2.4% in the sCSF1 K/O mice, and bone loss was negligible at femur while the control animals lost >11.2% of their bone mass at that site. Interestingly, total-body BMD actually increased in the K/O animals while declining significantly in the controls.

Currently available therapies for estrogen-deficiency bone loss include bisphosphonates, raloxifene and PTHRI-agonists. While these agents are effective at attenuating estrogen-deficiency bone loss, all of them have side effects, and so there remains a need for new therapies to maintain bone health in older women. As noted, preclinical data suggest that CSF1 may be a viable therapeutic target, since neutralizing CSF1 *in vivo* prevented OVX-induced bone loss.^26^ The availability of neutralizing antibodies to CSF1, which are currently in clinical trials for cancer and bone metastases,^42^ when coupled with the data presented here, suggest that CSF1 may be a reasonable target for drug discovery in treating menopausal bone loss.

In summary, the current findings, along with our earlier published work,^29,41^ indicate important non-redundant functions for the two isoforms of CSF1. Expression of mCSF1 is essential for normal skeletal metabolism since, in its absence, bone mass is significantly increased. sCSF1 is not required for normal skeletal acquisition, but in its absence the effect of estrogen withdrawal on the skeleton is markedly attenuated. Coupled with the findings alluded to earlier regarding a possible role for sCSF1 in inflammatory arthritides, our findings suggest that sCSF1 may participate in several states of disordered skeletal homeostasis.

## Figures and Tables

**Table 1:** Measurements of sCSF1 and mCSF1 protein levels in sCSF1 K/O mice

Proteins	*N*	Wt	K/O
Serum sCSF1/(pg·mL^−1^)	40	1052±52	None detected
Cell-associated mCSF1 (pg·mg^−1^ membrane protein)	10	202±26	160±18

K/O, knockout; Wt, wild type.

**Table 2:** Histomorphometry in sCSF1 K/O and Wt-OVX mice

Mice types	*N*	(Obs/BS)/%	(Obs/OS)/%	(NOb/TAR)/mm^−2^	(Nob/BPm)/mm^−2^	(Ocs/BS)/%	(NOc/TAR)/#	(NOc/BPm)/mm^−2^
sCSF1 K/O OVX	6	6.77±1.05	54.58±2.28	62.66±11.38	6.54±1.06	4.30±0.93	10.84±1.57	1.28±0.26
Control OVX	6	8.32±1.22	51.54±1.26	59.22±8.59	7.97±1.11	15.22±2.4	34.82±5.04	4.62±0.61
*P*		0.36	0.29	0.81	0.37	<0.001	<0.001	<0.001
